# Nanomechanics of negatively supercoiled diaminopurine-substituted DNA

**DOI:** 10.1093/nar/gkab982

**Published:** 2021-10-29

**Authors:** Domenico Salerno, Claudia Adriana Marrano, Valeria Cassina, Matteo Cristofalo, Qing Shao, Laura Finzi, Francesco Mantegazza, David Dunlap

**Affiliations:** School of Medicine and Surgery, BioNanoMedicine Center NANOMIB, Università di Milano-Bicocca, via R. Follereau 3, Vedano al Lambro (MB), Italy; School of Medicine and Surgery, BioNanoMedicine Center NANOMIB, Università di Milano-Bicocca, via R. Follereau 3, Vedano al Lambro (MB), Italy; School of Medicine and Surgery, BioNanoMedicine Center NANOMIB, Università di Milano-Bicocca, via R. Follereau 3, Vedano al Lambro (MB), Italy; School of Medicine and Surgery, BioNanoMedicine Center NANOMIB, Università di Milano-Bicocca, via R. Follereau 3, Vedano al Lambro (MB), Italy; Department of Physics, Emory University, Atlanta, GA USA; Department of Physics, Emory University, Atlanta, GA USA; School of Medicine and Surgery, BioNanoMedicine Center NANOMIB, Università di Milano-Bicocca, via R. Follereau 3, Vedano al Lambro (MB), Italy; Department of Physics, Emory University, Atlanta, GA USA

## Abstract

Single molecule experiments have demonstrated a progressive transition from a B- to an L-form helix as DNA is gently stretched and progressively unwound. The particular sequence of a DNA segment defines both base stacking and hydrogen bonding that affect the partitioning and conformations of the two phases. Naturally or artificially modified bases alter H-bonds and base stacking and DNA with diaminopurine (DAP) replacing adenine was synthesized to produce linear fragments with triply hydrogen-bonded DAP:T base pairs. Both unmodified and DAP-substituted DNA transitioned from a B- to an L-helix under physiological conditions of mild tension and unwinding. This transition avoids writhing and the ease of this transition may prevent cumbersome topological rearrangements in genomic DNA that would require topoisomerase activity to resolve. L-DNA displayed about tenfold lower persistence length than B-DNA. However, left-handed DAP-substituted DNA was twice as stiff as unmodified L-DNA. Unmodified DNA and DAP-substituted DNA have very distinct mechanical characteristics at physiological levels of negative supercoiling and tension.

## INTRODUCTION

The conformation and flexibility of DNA depend on electrostatics, base pairing, and base stacking. These interactions produce sequence specific characteristics that influence topology and protein binding. Denaturation directly reflects the hybridization of DNA but does not reveal manifold conformational dynamics of the base-paired state. In addition, there is a continuum of flexural and torsional DNA rigidity conferred by sequence, oxidative damage, or physiological modifications like methylation that alter the electrostatics, aromatic overlaps, or hydrogen bonding of base pairs (1). More constitutive modifications of DNA structure also exist. For example, 2, 6-diaminopurine (DAP) is an alternative nucleobase that can substitute adenine in A:T base pairs to add an exocyclic amine moiety in the minor groove (2). This amine group forms an additional hydrogen bond with the C2 carbon of thymine in a DAP:T base pair. Complete substitution of adenine- with DAP-deoxyribonucleotide triphosphate in polymerase chain reactions produces triple hydrogen bonding throughout the amplicon. Many years ago, a cyanophage with this change was reported (3), and just this year, DAP-substituted DNA (DAP DNA) genomes were found in dozens of other phages and several bacteria (4). DAP may be useful to probe ligand-DNA interactions (5) as well as DNA structure and the activity of enzymes that modify DNA topology or process genetic information (6,7), since the substitution of DAP for adenine changes base stacking and hydrogen bonding without altering the charge along the molecule.

Substitution of DAP for adenine is known to increase the melting temperature of the B-form (1,8,9). Sequence-specific effects persist and the Santa Lucia model for calculating the melting temperature (10) can be adjusted by uniformly scaling the dinucleotide enthalpies (11). Other data regarding the cyclization of DAP DNA fragments or binding and obligate tight curling around histone octamers to form nucleosomes indicates that DAP DNA is flexurally stiffer than unmodified DNA (6,12,13). This is substantiated by increased stiffness exhibited by DAP DNA molecules a few hundred- to kilo-bases in length deposited on surfaces for atomic force microscopy (6,11,13) and the increased persistence lengths determined using optical or magnetic tweezers to stretch single molecules of DAP DNA (11,13). Positively supercoiled DAP DNA under slight tension also exhibits increased flexural stiffness associated with plectonemic coils (7). While the persistence length of DAP DNA reflects increased rigidity, the overstretching transition of this DNA occurs at a lower tension (11). Thus, DAP DNA is more rigid and also more susceptible to twist-induced, structural, phase changes.

Perhaps the most striking phase change exhibited by DAP DNA is the conversion from a right- to a left-handed helical form. Although DAP-substitution maintains the charge density, the molecule adopts an unusual X-DNA conformation in response to high salt or ethanol concentrations (14). This has been shown to be a left-handed helical form with a zigzag phosphate backbone due to alternating syn- and anti-deoxyribose orientations (15). Circular dichroism studies of oligonucleotides of dGC repeats show that high salt concentrations, approximately 60% solutions of alcohols, or added nickel drive the formation of left-handed Z-DNA (15,16). Critical to this conformational change is the dehydration of the minor groove. Substituting diaminopurine for adenine has a similar effect by inserting an exocyclic amine that displaces water from the minor groove, but in an appropriate solvent stabilizes the X-DNA form, which displays a highly negative CD signal at 280 nm (14). The fact that X-form DAP DNA is recognized by Z-DNA antibodies and that P^31^ NMR studies show phosphate signals similar to those recorded for poly(dAT) in an X-DNA form indicate a zigzag backbone.

The exocyclic amine group introduced by DAP substitution not only creates a third Watson and Crick hydrogen bond to thymine, it also includes a non-planar hydrogen that can establish inter-base pair hydrogen bonding. Compared to intra-base pair hydrogen bonding, H-bonds between adjacent base pairs could more directly torque propeller twist angles of base pairs and more strongly link successive base pairs to alter winding angles. Such base pairing is theoretically feasible and the total number of H-bonds (Watson and Crick and inter-base pair) correlates directly with thermal stability of the duplex (17). The number of possible inter-base pair hydrogen bonds correlates indirectly with the flexibility of base pair steps. Inter-base pair hydrogen bonding is a relatively new hypothesis that might explain how winding and twist angle could be correlated with 275 nm CD signals (18) from natural DNA versus DNA with diaminopurine or inosine substitutions (13). H-bonding is likely to be a significant part of enhanced base stacking that stiffens DAP-substituted DNA.

The first sequences shown to shift to a left-handed form were pCpG repeats in extremely high salt (19). Genomic DNA is more random than poly(dG-dC) and any Z-DNA that forms will necessarily differ from the purine-pyrimidine pattern. These interruptions may be the basis of kinks in left-handed DNA according to the crystal structure of Z-DNA reported by de Rosa et al. (20) for a CG sequence with an intervening A:T base pair. They co-crystalized the Z-forming (CG)_3_A(CG)_3_ helix with Z-DNA binding Zα protein and found a 20–27 degree kink at the central A:T base pair. The plasmid sequences used in the work described herein may have short stretches of poly(dC-dG) that would form left-handed DNA when stretched and unwound into a left-handed conformation, but there would be many interruptions of the purine pyrimidine pattern where kinks might occur.

It is noteworthy that low levels of unwinding and tension have also been shown to drive the transition from right- to left-handed helices in GC segments of DNA (21), which suggested that triple hydrogen bonding may favor the right-to-left transition. To investigate this possibility, Magnetic Tweezers (MT) were used to gently stretch and unwind both unmodified and DAP-substituted DNA. MT is a well established single molecule tool for the simultaneous application of torsion and tension to a single DNA molecule (22–26). Changes in the extension of tethered molecules in response to twist, revealed differences in the nanomechanical characteristics of DAP-substituted versus unmodified DNA. In this work, the measurements were performed with two DNA filaments of different lengths, 4642 bp (DNA1) or 6258 bp (DNA2) sequences with 46 or 51% randomly distributed GC base pairs respectively. The DNA characteristics and the main results are summarized in Supplementary Table S1 of Supplementary Material. Within the experimental errors, we do not detect any systematic difference between the results obtained with the two different DNA sequences.

The experiments compared the progressive transition from the B- to L-form of DAP-substituted DNA or unmodified DNA upon unwinding with MT. The transition to left-handed forms of both DAP-substituted and unmodified DNA occurred at low levels of unwinding and tension. Tenfold lower persistence lengths compared to B-forms indicate a wide range of conformations are available to DNA in physiological conditions. Measurement of the extension versus twist curves indicated a 40% greater axial rise for the L-form of both DAP and unmodified DNA, with respect to their B-forms. Like the corresponding B-form configurations, left-handed DAP DNA was significantly stiffer than unmodified L-DNA. From an analysis of the progressive phase change from right- to left-handed helices, the persistence length of left-handed DAP DNA is calculated to be about twice that of left-handed unmodified DNA.

## MATERIAL AND METHODS

### Preparation of DAP and WT DNA

The DAP-substituted or unmodified DNA1 constructs were prepared as previously described (11). Briefly, for the MT experiments T7 DNA ligase was used to ligate a ∼1.0 kbp, multiply digoxigenin-labeled (dig-tail) DNA fragment at one end of main DNA fragment and a ∼0.9 kbp, multiply biotin-labeled (bio-tail) DNA fragment to the other end.

The DAP-substituted or unmodified DNA2 constructs consist of a three-component assembly: a 6258 bp core fragment, and two flanking tails containing biotin-16-dUTPs or digoxigenin-11-dUTPs, both 520 bp long. The core fragment was obtained by PCR using the Long Amp^TM^ (New England Biolabs) polymerase, the pL6 plasmid as template and the primers 5′ ACACCCGGGACCAGGACGCAGATATAGCC and 5′ CTAGGGCCCGCCGTAGAAGAACAGCAAGG, which contain XmaI and ApaI restriction sites (underlined), respectively. The DAP-substituted DNA was produced replacing dATP with 2,6-diaminopurine-5′-triphosphate (Trilink Biotechnologies, San Diego, CA) at the same concentration (200μM) as the other three dNTPs (Fermentas,Waltham, MA). The biotin and digoxigenin-labeled tails fragments were amplified by PCR using Taq polymerase in standard buffer (NEB) from the pBR322 plasmid. PCR solutions were supplemented with biotin-11-dUTP (Fermentas) and digoxigenin-11-dUTP (Roche, Indianapolis, IN) in a 1:4 ratio with respect to dTTP. The biotin-labeled tail was amplified using the forward primer 5′ GGAGTCCCGGGCAGAGTTCTTGAAGTGGTGG which contain the XmaI restriction site (underlined) and the revers primer 5′ GGTCCAGTCGTCGGGTCTCGCGGTATCATTGC. The digoxigenin-labeled tail was amplified using the forward primer 5′ GCTTGGGCCCCAGAGTTCTTGAAGTGGTGG which contains the ApaI restriction site (underlined) and the same reverse primer of the biotin-labeled tail. Following PCR amplification and column purification (Qia Quick PCR cleanup; Qiagen) the core fragment and the tails were digested with ApaI and XmaI restriction enzymes (New England Biolabs), purified again, and concentrations were measured by UV absorption. To assemble the final tether, from 500 to 1500 ng of each core fragment were mixed with both tails in a molar ratio of 1:3 along with 400 units of T4 DNA ligase (NEB) in a total volume of 60 μL and incubated at 16°C for 24 hours. The success of the ligation was verified by 0.8% agarose gel electrophoresis.

### Magnetic Tweezers setup and measurements

The custom made MT setup was previously described (27–30), and consisted of an inverted optical microscope equipped with an oil-immersion objective (NIKON 100x, NA = 1.25) mounted on a piezoelectric focusing system (PIFoc, Physik Instrumente, Bresso, Italy). The objective, coupled with a 15 cm focal-length lens, led to a 75x magnification. The magnetic field was generated by two permanent neodymium magnets placed above the flow chamber and two piezoelectric motors controlled the position of the magnets along the optical axis (z-direction) and the rotation around the same axis in order to apply a stretching force, or a torque, to the torsionally constrained DNA.

### Microchamber preparation

The flow cell consisted of a square glass capillary tube (1 × 1 mm^2^ section, 5 cm long, VitroCom, Mountain Lakes, NJ). For each measurement, 250 μl of DNA and magnetic beads suspension were injected, in the absence of a magnetic field, into the capillary tube previously functionalized as follows. First, a solution of 100 mg/ml polystyrene (average MW230000, Sigma Aldrich) in toluene was injected into the capillary. Then, the capillary was drained and dried with compressed air. In this way, the internal walls were uniformly coated with polystyrene (31). Next, 5 μg of sheep polyclonal anti-digoxigenin antibody (Roche) in 100 ml with 10 mM PBS were incubated in the capillary for 2 h at 37°C. Unbound anti-digoxigenin was eliminated by rinsing the capillary tube with PBS-Tween-20. The functionalized surface was passivated for 2 h at 37°C with a solution consisting of 10 mM PBS at pH 8 supplemented with 0.1% Tween-20, 1 mg/ml fish sperm DNA (Roche) and 3 mM NaN_3_ (32). Finally, the capillary tube was rinsed with PBS-Tween-20 and incubated for 1h to allow the DNA to bind to the lower capillary tube surface. For storage, several capillaries could be prepared simultaneously and kept at 20°C after air drying them.

### Data acquisition and analysis

Images were acquired by a CCD camera (Marlin Allied vision, USA) running at a frame rate of 60 Hz and analyzed in real time by a custom software developed in Java (Oracle, USA). Calibration of the diffraction ring profiles of the magnetic beads was used to measure the 3D position of the beads with a precision of 10 nm along the optical axes and 40 nm in x-y plane (27). The extracted data was recorded for successive analysis that was performed with an *ad hoc* software written in MatLab (Mathwork inc. USA).

## RESULTS

### The torsional behavior of unmodified and DAP DNA

**Figure 1.**
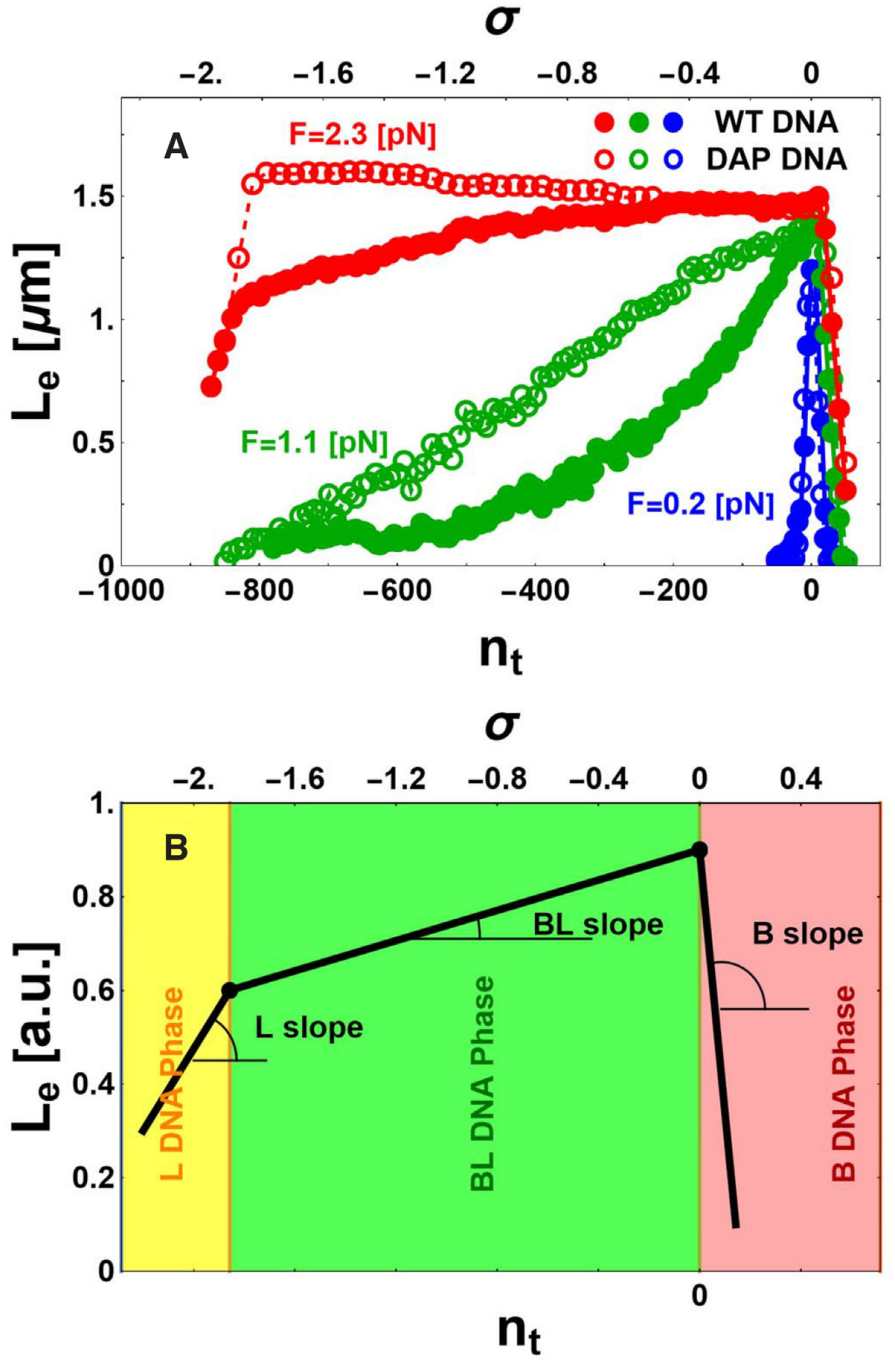
Torsional behavior of WT and DAP DNA. (**A**) Representative MT data for WT (filled disks) and for DAP (open circles) DNA display different extensions L_e_ measured as a function of the number of imposed turns n_t_ (lower axis) or supercoiling density σ (upper axis) at fixed values of force F. (**B**) Simplified sketch of theoretical DNA extension L_e_, obtained as a function of n_t_ or σ at fixed values of F. The different, color-coded DNA phases are: L (yellow), mixed BL (green), and B (red). The specified angles illustrate the different slopes dL_e_/dn_t_ characterizing the L, BL, and B phases.

In this work, we studied the difference between the nanomechanical behavior of unmodified (WT) and DAP DNA in conditions of high negative torsion. Representative twist experiments are reported in Figure 1A where the DNA extension L_e_ is measured as a function of turns of the bead attached to the DNA, n_t_, or the corresponding supercoiling, σ = n_t_/(N_b_/10.4), where N_b_ = 4642 is the number of base pairs of the DNA construct. Figure 1A shows data acquired over a large range of imposed turns (from n_t_ = +100 until n_t_ = -900 turns) for WT and DAP DNA at three different forces F (0.2, 1.1, or 2.3 pN). Additional data at different forces are reported in Supplementary Figure S1. Whereas at low forces (F = 0.2 pN), the behaviors of WT and DAP DNA were basically indistinguishable, at intermediate (F = 1.1 pN) and high (F = 2.3 pN) forces, the two types of DNA show qualitatively and quantitatively different characteristics. In particular, at intermediate and high forces the extension of unwound DNA appeared systematically higher for DAP DNA than for WT DNA. Furthermore, for high forces (F = 2.3 pN) three different linear regimes are clearly distinguishable.

These three regimes of typical L_e_ vs n_t_ data are schematically diagrammed in Figure 1B. The data compose three linear segments, where L_eB_, L_eBL_, and L_eL_ define the DNA extension of the B, BL, and L forms (BL indicates a mixture of B and L forms). Each segment is characterized by a slope, dL_e_/dn_t_. For n_t_ > 0 (red region), the DNA remains in the B-form and dL_eB_/dn_t_ is negative. For intermediate negative values of n_t_ (green region), the DNA is a mixed phase of B- and L-forms and, depending on the force, extension either decreases or increases as a function of n_t_, corresponding to shallow positive or negative BL slopes, dL_eBL_/dn_t_, respectively. For higher negative values (σ < -1.8; yellow region), the DNA is in the L-form and dL_eL_/dn_t_ is positive and steep.

Based on these types of observations, slopes in the B, BL and L regimes were plotted as a function of force for WT and DAP DNA.

#### B-form DNA

**Figure 2.**
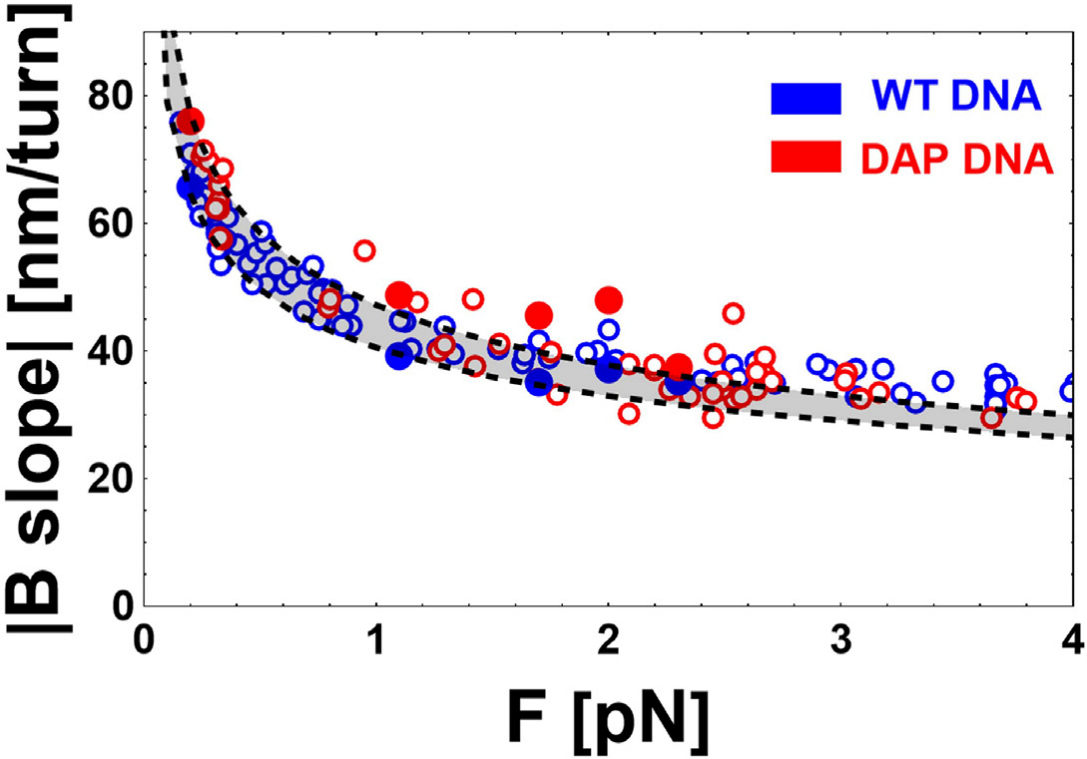
Force dependence of the slope of the extension versus n_t_ data for B-form of WT and DAP DNA. Experimental values (dots) and theoretical predictions (dashed black curves) for the slope dL_eB_/dn_t_ of WT (blue) and DAP-substituted (red) DNA, for DNA1 (filled dots) and DNA2 (open dots). Slopes are given in absolute values. Predictions from the model (40) described in the text assumed: I_s_= 150 mM and L_pB_= 30 nm (lower line) or L_pB_= 70 nm (upper line). A rather broad range of persistence lengths well represents either data set (shaded region).

As shown in Figure 2, the absolute values of the slopes dL_eB_/dn_t_, calculated for the (B and n_t_ > 0) region of the experimental L_e_ vs n_t_ curves decrease with force. Slopes for DAP DNA are not significantly steeper than those of unmodified DNA, while fits of the Worm Like Chain (WLC) (33) to the data produced persistence length estimates of 48.2 ± 6.8 nm and 44.3 ± 7.4 nm for DAP and WT, respectively.

#### BL-form DNA

**Figure 3.**
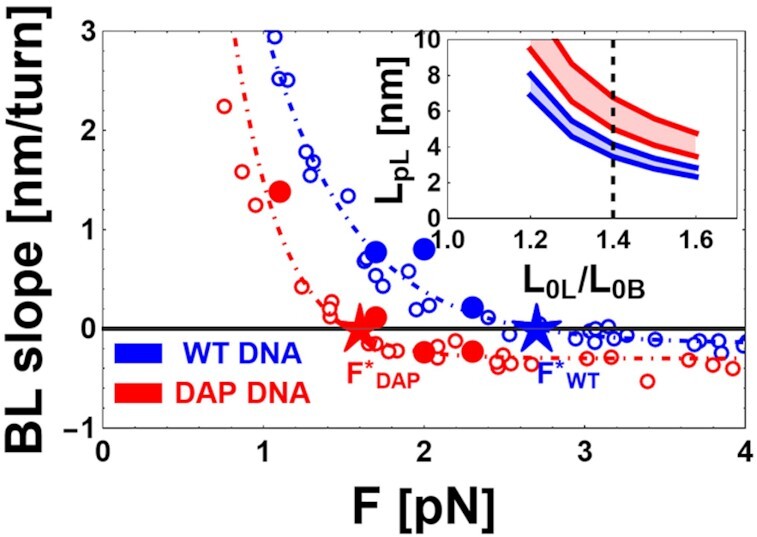
Force dependence of the slope of the BL phase for WT and DAP DNA. Main panel: experimental data (dots) for the slope dL_eBL_/dn_t_ of the BL-DNA phase are shown as a function of the applied force F for WT (blue) and DAP (red) DNA for DNA1 (filled dots) and DNA2 (open dots). The dot-dashed lines are a guide for the eye. F*_DAP_= 1.6 pN and F*_WT_= 2.7 pN (stars), represent the measured inversion force for DAP and WT DNA, respectively. Inset: theoretical predictions of the regions of the values of L_pL_ and L_0L_/L_0B_ compatible with the measured values of the inversion forces (red region for F*_DAP_ ± 0.3 pN and blue region for F*_WT_ ± 0.3 pN, respectively). The vertical dashed line represents the assumed values of L_0L_/L_0B_ for WT and DAP (see text for details).

The slopes calculated in the intermediate BL region, dL_eBL_/dn_t_, are shown in Figure 3. In this case the extension versus turns data of DAP DNA have significantly flatter slopes than those of WT DNA. Furthermore, for both types of DNA, slopes in the BL region become zero at particular values of force, which was defined as the inversion force F*, marked by stars in Figure 3, where the BL slope is zero. The inversion force is higher for WT DNA (F*_WT_ = 2.7 ± 0.3 pN) than for DAP DNA (F*_DAP_ = 1.6 ± 0.3 pN). When the DNA is in the BL-form, at high forces the L_e_ vs n_t_ data are approximately linear. At lower forces, a non-zero probability of plectonemic formation can alter the measured slope. However, the BL slope was flat and displayed no plectoneme formation at the measured inversion forces.

#### L-form DNA

**Figure 4.**
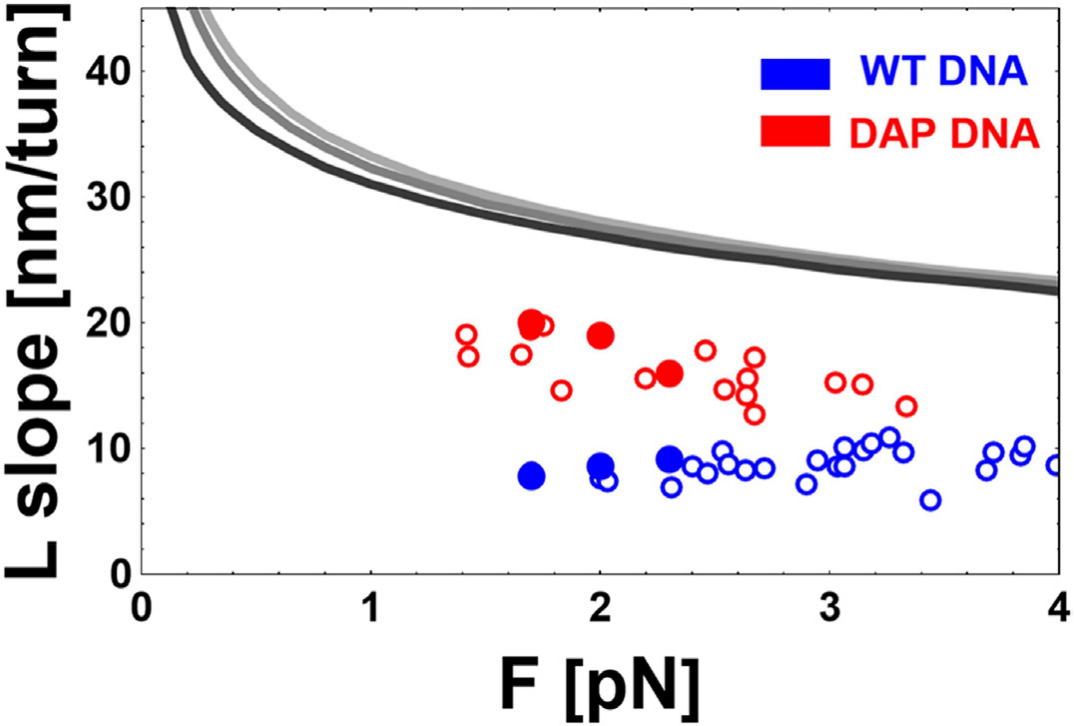
Force dependence of the experimental data (symbols) and the theoretical predictions of the model (40) (gray continuous lines) for the slopes dL_eL_/dn_t_ of the L-phase for WT (blue) and DAP (red) DNA. Data correspond to DNA1 (filled dots) and DNA2 (open dots). Parameters used in the model: ionic strength I_s_= 150 [mM] and increasing values of L_pL_= 1, 4, 6 nm, from dark to light shades of gray. (See Discussion).

Finally, Figure 4 shows the dependence of the L slopes dL_eL_/dn_t_ (L slopes) on the applied force for both WT and DAP DNA. The magnitudes of L slopes are smaller with respect to the corresponding B slopes. Furthermore the L slopes for DAP appear significantly higher than those for WT DNA.

### Theoretical analysis

The variation of the DNA extension under an imposed torsion is due to relaxation of the torsion by forming plectonema and/or denaturation bubbles (34). For small applied forces (F < 0.5 pN), the DNA is in B-form and the formation of plectonema is strongly favoured, consequently the variations in DNA extension as a function of torsion are significant. At negative and intermediate values of n_t_ or σ (-800 < n_t_ < -50; -1.8 < σ < -0.1) and higher values of applied force (F > 1 pN), i.e. in the DNA BL-form regime, the formation of L-form DNA induces slight variations in the DNA extension and the number of base pairs involved in such transition is proportional to n_t_ or σ (35). For large n_t_ or σ absolute values (n_t_ < -800; σ < -1.8) the DNA is completely denatured, its conformation changes from B to L, and any further decrement of n_t_ or σ likely induces the formation of plectonema in segments of the L-form DNA.

In the following, we propose a simple theoretical explanation for understanding the torsional behavior of the three DNA forms discussed thus far in either WT or DAP DNA.

#### B-form DNA

The variations of the DNA extension under positive imposed turns, or negative turns at low forces, is due to the formation of plectonema (36–38). Precise modeling of the geometrical structure of such plectonema has been already proposed, and it successfully predicts quantitatively the B slope of experimentally measured L_e_ vs n_t_ curves in WT DNA (39–42). Those authors extracted the geometry of plectonema, which minimized the total energy by calculating the total energy as the sum of the potential, bending, and electrostatic energies (40). The resulting prediction for the B slope according to the model of (40) are represented by the dashed black curves in Figure 2, calculated at 150 mM NaCl for persistence lengths L_p_ = 30 nm and L_p_ = 70 nm. These L_pB_ values have been chosen to outline the sensitivity of the model and experiments. Obviously, a broad range of values of L_p_ are compatible with the data (see Discussion). Including experimental uncertainties, the slopes of B regions for WT or DAP DNA are indistinguishable. In (7) and (11), the L_p_ and L_0_ values were obtained by fitting the WLC model to the DNA extension as a function of the applied force at zero imposed turns. In Supplementary Figure S4A and S4B, we report a set of new data for DNA2. The resulting fitting parameters (contour length, L_C_ and persistence length, L_p_) are L_CB,WT_ = 2.0 ± 0.2 μm, L_pB,WT_ = 44.3 ± 7.4 nm, L_CB,DAP_ = 2.1 ± 0.1 μm, L_pB,DAP_ = 48.2 ± 6.8 nm compatible with the number of DNA base pairs and with the results reported in literature (7,11).

#### BL-form DNA

By further decreasing σ, especially in DNA molecules under increased tension, the DNA extension is well described by a combination of B and L-DNA (mixed BL phase) (39,43). Standard B-DNA is characterized by a persistence length, L_pB_ ≈ 50 nm, and a distance between base pairs L_0B_ = 0.34 nm. The same parameters are generally difficult to estimate for L-DNA, but a value of L_pL_ ≈ 3 nm and a corresponding increment of the extension L_0L_/L_0B_ = 1.41 have been reported for WT DNA (39). Overall, L-DNA appears to be more flexible and elongated with respect to B-DNA. The percentage of L-DNA induced by torsion increased linearly with σ, until the critical value of n_t,max_ ≈ -800, or σ_max_ ≈ -1.8, where all B-DNA was converted to L-DNA. At the two extremes (n_t_ = 0 or σ = 0 and n_t,max_ ≈ -800 or σ_max_ ≈ -1.8), the DNA extension, L_eB_ and L_eL_, respectively, is accurately modelled as a function of the external force F, by WLC (33,44,45) with the corresponding L_0B_, L_pB_ and L_0L_, L_pL_ for pure B and L-DNA, respectively (46). The relation between F and both L_eB_ and L_eL_ is described by the function *f* given by the classical WLC model as follows:

(1) $$\begin{eqnarray*} F &=& f \left( {{L_{eB}},{L_{pB}},{L_{0B}}} \right) \nonumber \\ &=& \frac{{{k_B}T}}{{{L_{pB}}}} \left( { - \frac{1}{4} + \frac{1}{{4 {{\left( {1 -\frac{{{L_{eB}}}}{{{N_b}{L_{0B}}}}} \right)}^2}}} + \frac{{{L_{eB}}}}{{{N_b}{L_{0B}}}}} \right) \end{eqnarray*}$$

(2) $$\begin{eqnarray*} F &=& f \left( {{L_{eL}},{L_{pL}},{L_{0L}}} \right) \nonumber \\ &=& \frac{{{k_B}T}}{{{L_{pL}}}} \left( { - \frac{1}{4} + \frac{1}{{4 {{\left( {1 -\frac{{{L_{eL}}}}{{{N_b}{L_{0L}}}}} \right)}^2}}} + \frac{{{L_{eL}}}}{{{N_b}{L_{0L}}}}} \right) \end{eqnarray*}$$

From which, it is possible to calculate the DNA extension as ${L_{eB}} = {f^{ - 1}} ( {F,{L_{pB}},{L_{0B}}} )$ and ${L_{eL}} = {f^{ - 1}}{\rm{ }}( {F,{L_{pL}},{L_{0L}}} )$.

At intermediate σ values the DNA extension L_eBL_ of the BL mixed phase can be described as a linear combination of the WLC contribution from the two pure phases B and L (see Supplementary for details):

(3) $$\begin{eqnarray*} {L_{eBL}} &=& {L_{eB}}{\rm{ }}\left( {1{\rm{ }} - \chi } \right){\rm{ }} + {\rm{ }}{L_{eL}}\chi {\rm{ }} = {\rm{ }}{f^{ - 1}}\left( {F,{L_{pB}},{L_{0B}}} \right)\left( {1{\rm{ }} - \chi } \right){\rm{ }} \nonumber \\ && +\, {f^{ - 1}}\left( {F,{L_{pL}},{L_{0L}}} \right)\chi \end{eqnarray*}$$

where χ represents the fraction of L-DNA present in the sample and it can be evaluated as

(4) $$\begin{equation*}\chi = \frac{{{n_t} - {n_{b,max}}}}{{{n_{t,max}} - {n_{b,max}}}} = \frac{{\sigma - {\sigma _{b,max}}}}{{{\sigma _{max}} - {\sigma _{b,max}}}} \end{equation*}$$

where n_b,max_ or σ_b,max_ are the number of turns, or supercoiling, corresponding to the maximum value of the turns relaxed by the DNA twisting, i.e. the maximum value of the buckling transition. The buckling transition increases with the applied force, but it reaches a maximum since it is limited by the presence of the B- to L-form transition (35). The absolute value of n_b,max_ is between 20 and 30 for the DNA lengths used in this manuscript and n_b,max_ is negligible with respect to the total imposed twist in the BL region. n_t,max_ or σ_max_ are the number of turns, or supercoiling, necessary for a complete conversion to the L-form at higher force (σ_max_ = n_t,max_/(N_b_/10.4)). Initially, when we increase |n_t_|, the DNA extension remains constant and torque accumulates and for 0 < n_t_ < n_b,max_ the DNA remains in the B form. After a certain number of turns, at low force the system buckles and a loop is formed. Upon further twisting, the helix coils upon itself forming intertwined loops and reduces the extension. Negative supercoiling at higher forces induces the formation of L DNA. In this regime, an additional twist raises the percentage of L DNA until the value of n_t_ = n_t,max_, where all the DNA is in the L form. Above this supercoiling value, the L DNA forms plectonemes, which contract the tether and produce positive dL_eL_/dn_t_ slopes.

**Figure 5.**
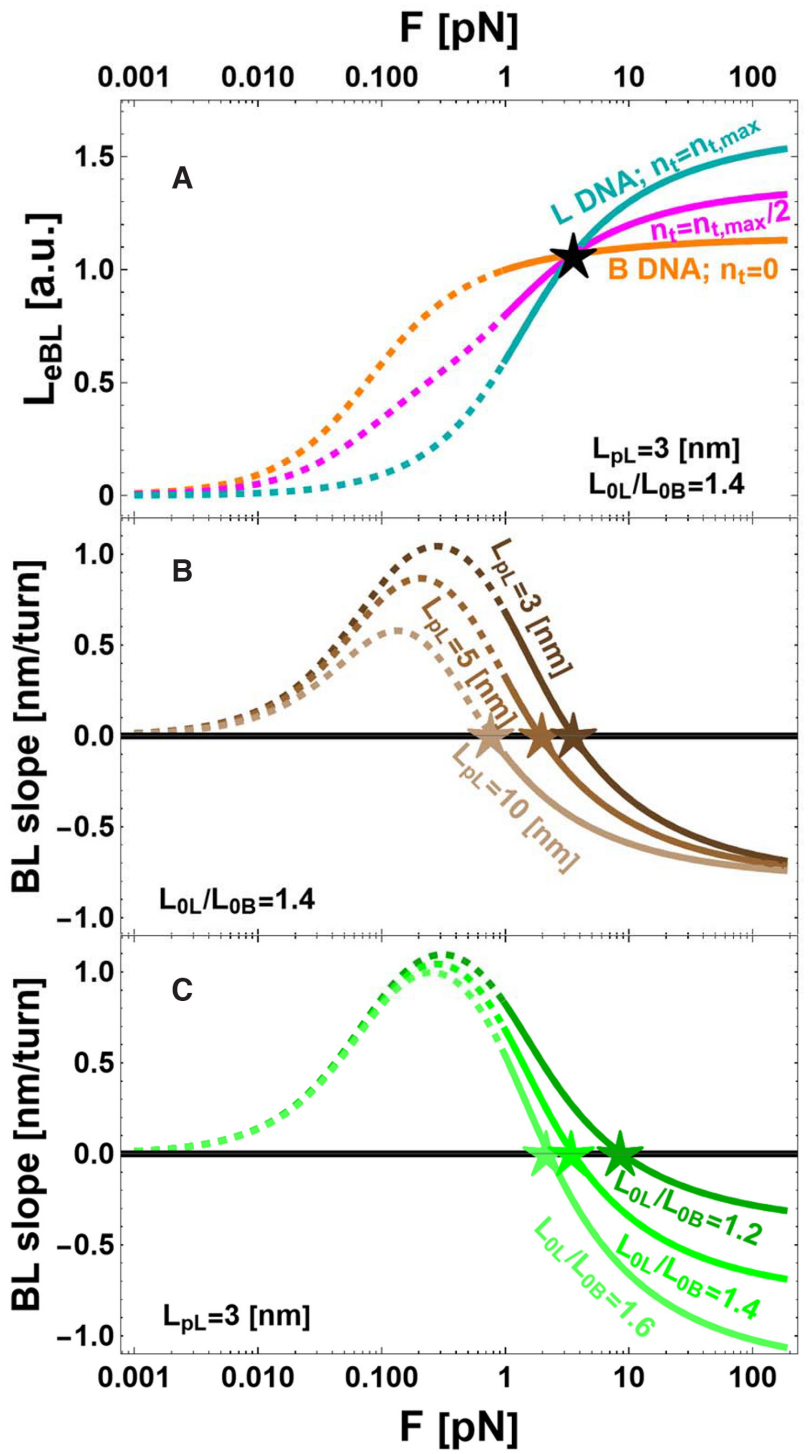
Simulations of DNA extension L_eBL_ and BL-phase slope, dL_eBL_/dn_t_, calculated according to the model described in the text. The theoretical predictions of the model are presented as dashed lines (outside the range of validity) or continuous lines (inside the range of validity). The stars indicate the forces at which extensions of B and L phases are identical, i.e. the inversion forces. (**A**) The calculated DNA extension L_eBL_ is plotted as a function of the applied force F with persistence length of the L phase, L_pL_= 3 nm, and ratio between the extension of L- and B-form DNA, L_0L_/L_0B_= 1.41, for various n_t_ (n_t_= 0 (orange); n_t_= n_t,max_/2 (magenta); n_t_= n_t,max_ (cyan)). The curves were obtained assuming L_0B_ = 0.34 nm, L_0L_ = 0.48 nm, L_pB_ = 50 nm, and L_pL_ = 3 nm. (**B**) The calculated BL-phase slope is plotted as a function of force with fixed L_0L_/L_0B_= 1.41 at L_pL_= 3 nm; L_pL_= 5 nm; L_pL_= 10 nm. (**C**) The calculated BL-phase slope is plotted as a function of force with fixed L_pL_= 3 nm at L_0L_/L_0B_= 1.2; L_0L_/L_0B_= 1.4; L_0L_/L_0B_= 1.6.

The results of this heuristic model are represented in Figure 5A, where the WLC predictions for L_eBL_*vs* F are shown in the case of the pure B (orange) and L (cyan) phases. The two limiting cases of pure B and L phases correspond to the values of n_t_ = 0 and n_t_ = n_t,max_, respectively, which are associated with values of χ ≈ 0 or χ ≈ 1 in Equation (4), assuming a negligible value of n_b,max_. In Figure 5A we show the predictions of the mixed WLC model for B and L DNA. As apparent from the Figure, introducing an increment of the contour length and a reduction of the persistence length from B to L DNA have a significant impact on the force extension curve. Indeed, the B-form is characterized by both a lower value of the asymptotic extension or contour length (L_0B_ < L_0L_) and a higher value of the persistence length with respect to the L-form (L_pB_ > L_pL_). As a consequence of the values of contour lengths of B and L DNA, the asymptotic value of the DNA extension at high forces is lower for the B-form with respect to the L-form, since the base pair separation is larger for L DNA with respect to B DNA (L_0B_ = 0.34 nm; L_0L_ = 0.48 nm). On the other side, at intermediate force values, a longer DNA extension is expected for the B-form with respect to the L-form, as a consequence of the higher entropic forces generated by the L DNA with shorter persistence length with respect to B DNA (L_pB_ ≈ 50 nm; L_pL_ ≈ 4 nm). This model predicts a specific force (inversion force F*) where the opposite contributions of the contour and persistence length modifications are balanced and the total length of the DNA chain is unmodified for any percentage of B and L form. This point is easily detectable in the MT torsional experiment and appears as the force where the BL slope is zero. The black star (Figure 5A) indicates the force at which extensions of B and L phases are equivalent, i.e. the inversion force. See details of the calculation in the Supplementary.

For intermediate values of σ, this simple model predicts a behavior of L_eBL_*vs* F, as shown by the magenta curve obtained for n_t_ = n_t,max_/2 (Figure 5A). Furthermore, this model allows calculation of the value of the BL slope as dL_eBL_/dn_t_ (Figure 5B and C) taking the n_t_ derivative of Equation (3). In all the plots of Figure 5, the theoretical predictions of the model are presented as dashed lines (outside the range of validity) or continuous lines (inside the range of validity), to emphasize that the proposed simplified model is valid only for high force values, where we can disregard plectoneme formation. Outside the range of validity, the measured BL slope is larger with respect to the theoretical predictions, since plectoneme formation induces a steeper change of the DNA extension. Given the values of L_0B_ and L_pB_, the free parameters of the model are the persistence length L_pL_ and the extension L_0L_ of the L-form. Figure 5B shows the predicted values of the BL slopes when L_0L_ is a constant and L_pL_ is allowed to vary, and vice versa in Figure 5C. A key result of this model is the prediction of inversion forces F* (stars in Figure 5) under which the BL slopes are zero for different values of L_0L_, and L_pL_. These inversion forces have been consistently calculated for several conditions (Supplementary Figure S2) where we show F* obtained by keeping the L_0L_/L_0B_ ratio constant and varying L_pL_ (Supplementary Figure S2A), or by keeping L_pL_ constant and varying the L_0L_/L_0B_ ratio (Supplementary Figure S2B). Reciprocally, from the predicted inversion forces it is possible to estimate the values of L_0L_/L_0B_ and L_pL_ compatible with the experimentally measured values of F*_WT_ = 2.7 ± 0.3 pN and F*_DAP_ = 1.6 ± 0.3 pN, which are indicated by the continuous horizontal lines (blue or red) in Supplementary Figure S2 together with their confidence range indicated by horizontal dashed lines. Indeed, by keeping constant the values of L_pB_ and L_0B_, it is possible to calculate a specific value of the inversion force by assuming the values L_pL_ and L_0L_. Reciprocally, it is possible to calculate the values of L_pL_ and L_0L_ compatible with a specific value of F*. Namely, the intersections of the horizontal lines of Supplementary Figure S2 with the calculated lines at various L_0L_/L_0B_ and L_pL_ indicates the L_pL_ and L_0L_/L_0B_ values compatible with the measured values of F*. The results are shown in the inset of Figure 3, where in the space of the two variables L_0L_/L_0B_ and L_pL_, we show the locus of values compatible with the range of measured values of the inversion force, represented as red and blue regions for DAP and WT, respectively.

#### L-form DNA

The response of L-form DNA to negative supercoiling is displayed at extreme negative values of the supercoiling (σ < -1.8). After extensive unwinding, the transition to L-form DNA is complete and any further torsion induces the formation of plectonemes. Since the physical phenomenon of plectoneme formation is presumably similar in L- and B-DNA, in principle it could be possible to apply the same model (40) used to explain the B slopes also for the L slopes. In Figure 4, we report the experimentally measured values (symbols) and the values of the L slope predicted by the model (continuous lines with different shades of gray) at its extreme range of validity i.e. obtained for very low values of persistence length (L_PL_ = 1, 4, 6 nm at I_S_ = 150 mM). The model qualitatively describes the data, since it predicts a mild decrease of the L slope with the force, but even for unjustifiably low values of L_pL_, the model fails to give a quantitative description of what is observed in the L-form for both WT and DAP DNA. (see Discussion).

## DISCUSSION

The experimental data presented here show significant similarities and differences between WT and DAP DNA. Both display a progressive transition from right to left-handed helices when unwound under intermediate tensions. Indeed, using MT it is possible to control the fraction of L-form, which facilitates the nanomechanical characterization of WT and DAP DNA in both the B and L forms. Such precise control is a critical feature of single molecule experimentation.

### B-form DNA

Firstly we note that, in the regime of positive supercoiling and negative supercoiling at low forces (<0.5 pN), both the WT and DAP-substituted DNA adopt the classical right-handed double helix with unperturbed hydrogen bonds between the Watson and Crick bases (DNA in the B-form). In these regimes, the slope of L_e_*vs*. number of imposed turns, n_t_, is due to the plectonemic geometry, which arises from B-DNA characteristics such as the persistence length and on the tension in the molecule (38–41,47). In the data presented here (Figure 2) we observe that the slopes for B-form DAP DNA are similar with respect to WT DNA. The prediction of the model we used (40 or https://home.uni-leipzig.de/mbp/index.php/software/) quantifies precisely the B slope dependence on the persistence length, force, and ionic strength. Accordingly, the dashed lines in Figure 2 show the slopes calculated following the model in (40), assuming values of persistence length L_p_ = 70 or 30 nm to illustrate the range compatible with our results and with what is reported in literature (7,11).

### BL-form DNA

The negative supercoiling region at higher forces (>1pN) exhibits significant differences between the BL and L slopes of WT and DAP DNA samples. The difference between the inversion force F* of DAP and WT DNA (Figure 3) is striking. This inversion force difference can be explained with the simplified model we obtained by combining in series a WLC model of B- and one of L-form DNA, each with its own characteristic L_p_ and L_0_ parameters.

Based on the assumption that the relative fraction χ of L-DNA is linearly dependent on the number of imposed turns, our model can predict persistence length L_pL_ and contour length L_0L_ values, which are derived from the measured inversion forces F*.

Our model is not sufficient to describe in more detail the L-form DNA mechanical parameters. Indeed, in principle, many pairs of values for L_pL_ and L_0L_/L_0B_ within the areas shown in the inset of Figure 3 satisfy the model. Nevertheless, it is possible to determine independently L_0L_/L_0B_ from our data with a simple geometrical reasoning. The argument is based on the observation that all the DNA phases share the same length of the backbone and the differences in the contour lengths are related to the helix geometrical properties. In particular, the DNA contour length is determined by the helical pitch that can be easily calculated from the threshold (σ = -1.8) at which the BL region ends. A simple calculation (see Supplementary Figure S3 for details) leads to a value L_0L_/L_0B_ = 1.4 equal for both WT and DAP, since both have the same threshold in σ = -1.8 for the BL to L transition. This value of 1.4 is also reported in the literature only for WT DNA (39,46). By using this value for L_0L_/L_0B_, we obtained the persistence length values L_pL,WT_ ≈ 3.8 nm and L_pL,DAP_ ≈ 6.0 nm for the L-form.

The resulting increment of the L-DNA persistence length of DAP with respect to WT DNA suggests a more structured and rigid form of the L phase in DAP DNA. This increment of DAP stiffness confirms the qualitative observation that DAP is more easily extended with respect to the WT at constant force (see Figure 1A). Unlike B-DNA, the helically intertwined but non-hybridized L-DNA form is not well defined. The L-form is related to the torsional stress that drives the two strands of DNA counterclockwise while the electrostatic repulsion of the backbone strands gives rigidity to the chain. Some DNA bases might experience large enough negative twist to allow base pairing in the left hand configuration. However, in this case, Z-DNA would result, being characterized by different mechanical parameters. In particular, for WT Z- DNA has a persistence length, L_pZ,WT_ = 200 nm, much higher than that of L-DNA (39,48). In the BL region we cannot exclude that a small fraction of Z-DNA phase might be present along the chain. Differing tendencies to generate Z-DNA in the BL region could explain the observed difference of L_pL_ determined for DAP DNA.

### L-form DNA

Finally, the measurements illustrated in Figure 4 shows a steeper dependence of L_eL_ on n_t_ for DAP DNA in the region where all the DNA chain is completely converted to L-DNA. In this region the slope is presumably due to the formation of plectonemes and an increment can be justified with an increase in the persistence length of the L-form. The difference in persistence length qualitatively explains the difference between dL_eB_/dn_t_ and dL_eL_/dn_t_, together with the difference between dL_eL,WT_/dn_t_ and dL_eL,DAP_/dn_t_. The model used to describe the plectonemes formation for the B-DNA (40) only qualitatively reproduces the behaviour of L-DNA. Indeed, as shown in Figure 4 the model predicts a mild decrease of the L-region slope with the force, but the measured slopes are less than one half of the predicted values. In the model, the DNA is represented as a flexible rod of specified radius and linear charge distribution, under the action of a force and torsion. L-DNA might be described by a smaller persistence length and the prediction for the L slope in this asymptotic regime is reproduced in Figure 4 by the gray lines obtained for low L_p_ values (L_p_ = 4 and 6 nm, which are the values suggested by the comparing model and data as shown in Figure 3 and L_p_ = 1 nm, which is the minimum conceivable value of the DNA persistence length). Even in this asymptotic regime, the measured L slope is too small with respect to the theoretical predictions for L slopes for both WT and DAP DNA. This discrepancy reflects the fact that the model fails beyond its range of validity and that a more refined approach is necessary. Assuming that the L slope simply depends on the formation of plectonema is likely naive, since as reported in (39) for the WT DNA, in the L regime the torque does not seem constant, as should be expected in a plectonemic regime. A possible way to reconcile all these clues is to assume the presence of a new DNA phase after the complete denaturation process. However, whatever the conformation of DNA in this region might be, the large difference between the L slopes attests to a significant difference in the mechanical parameters of DAP with respect to WT DNA in this extremely negatively coiled regime.

In conclusion, given the low levels of unwinding and tension at which L-DNA forms, it likely plays a role in a wide range of DNA transactions. The higher bending rigidity of DAP DNA compared to normal DNA, imposed larger plectonemic gyres at high levels of unwinding. These features emphasize the dependence of the mechanics of L-form DNA on the extent of triple hydrogen bonding and/or on modifications that alter the minor groove and the associated stabilizing spine of water molecules (49) or the major groove in which methylation makes DNA more susceptible to B-Z transitions (50). More generally, the different mechanical properties of DAP expand the tool kit for the rational design of biomaterials.

## DATA AVAILABILITY

All data is available upon request.

## Supplementary Material

gkab982_Supplemental_File

## Notes

Present address: Qing Shao, School of Science and Technology, Georgia Gwinnett College, Lawrenceville, GA (USA).
