# Supplementary material for: Nanomechanics of negatively supercoiled diaminopurine-substituted DNA
Source: Nucleic Acids Res. 2021 Oct 29;49(20):11778–86. doi: 10.1093/nar/gkab982 (PMC8599871; doi:10.1093/nar/gkab982)
Supplement: gkab982_Supplemental_File [file gkab982_supplemental_file.pdf]

Nanomechanics of negatively supercoiled diaminopurine-substituted DNA.

Domenico Salerno[1#], Claudia Adriana Marrano[1#], Valeria Cassina[1], Matteo Cristofalo[1], Qing Shao[2‡], Laura Finzi[2], Francesco Mantegazza[1,*], David Dunlap[2,*]

[1] School of Medicine and Surgery, BioNanoMedicine Center NANOMIB, Università di Milano-Bicocca, via R. Follereau 3, Vedano al Lambro (MB), Italy

[2] Department of Physics, Emory University, Atlanta, GA (USA)

[‡] Current address: School of Science and Technology, Georgia Gwinnett College, Lawrenceville, GA (USA)

[#] D. Salerno and C.A. Marrano contributed equally to this work.

* To whom correspondence should be addressed. Francesco Mantegazza Tel: +39-02-6448-8209; Email: francesco.mantegazza@unimib.it and David Dunlap Tel: +1-404-727-8036; Fax: +1 404-727-0873; Email: ddunlap@emory.edu

SUPPLEMENTARY DATA

	WT DNA (DNA1/DNA2)	DAP DNA (DNA1/DNA2)
N_b (number of base pairs)	4642/6258	4642/6258
percentage of triple H bonds between the DNA bases	46%/51%	100%/100%
$n_{t,max}$ (number of turns necessary for the complete conversion to L form)	≈800/≈1100	≈800/≈1100
$\sigma_{t,max}$ (supercoiling necessary for the complete conversion to L form)	≈1.8	≈1.8
L_{0B} (base pair distance in the B form)	0.34 nm (literature)	0.34 nm (reported in Reference 7 and 11)
L_{0L} (base pair distance in the L form)	0.48 nm = 1.41•L_{0B} (reported in Reference 39)	0.48 nm = 1.41•L_{0B} (calculated as discussed in Figure S3)
L_{pB} (persistence length in the B form)	44.3 ± 7.4 nm (calculated from WLC fitting on L_e vs F data as shown in Figure S4A)	48.2 ± 6.8 nm (calculated from WLC fitting on L_e vs F data as shown in Figure S4A)
L_{pL} (persistence length in the L form)	3.8 ± 0.4 nm (calculated from BL slopes and the inset of Figure 3)	6.0 ± 1.0 nm (calculated from BL slopes and the inset of Figure 3)
F* (Inversion force where $dL_{eBL}/dn_t = 0$)	2.7 ± 0.3 pN (obtained from BL slopes of Figure 3)	1.6 ± 0.3 pN (obtained from BL slopes Figure 3)

Table S1. Quantitative summary of the DNA characteristics and of the main results of the reported work. When in a cell table two numerical values are separated by a "/" symbol, they should be interpreted as the values of the two different filaments DNA1 and DNA2, respectively.

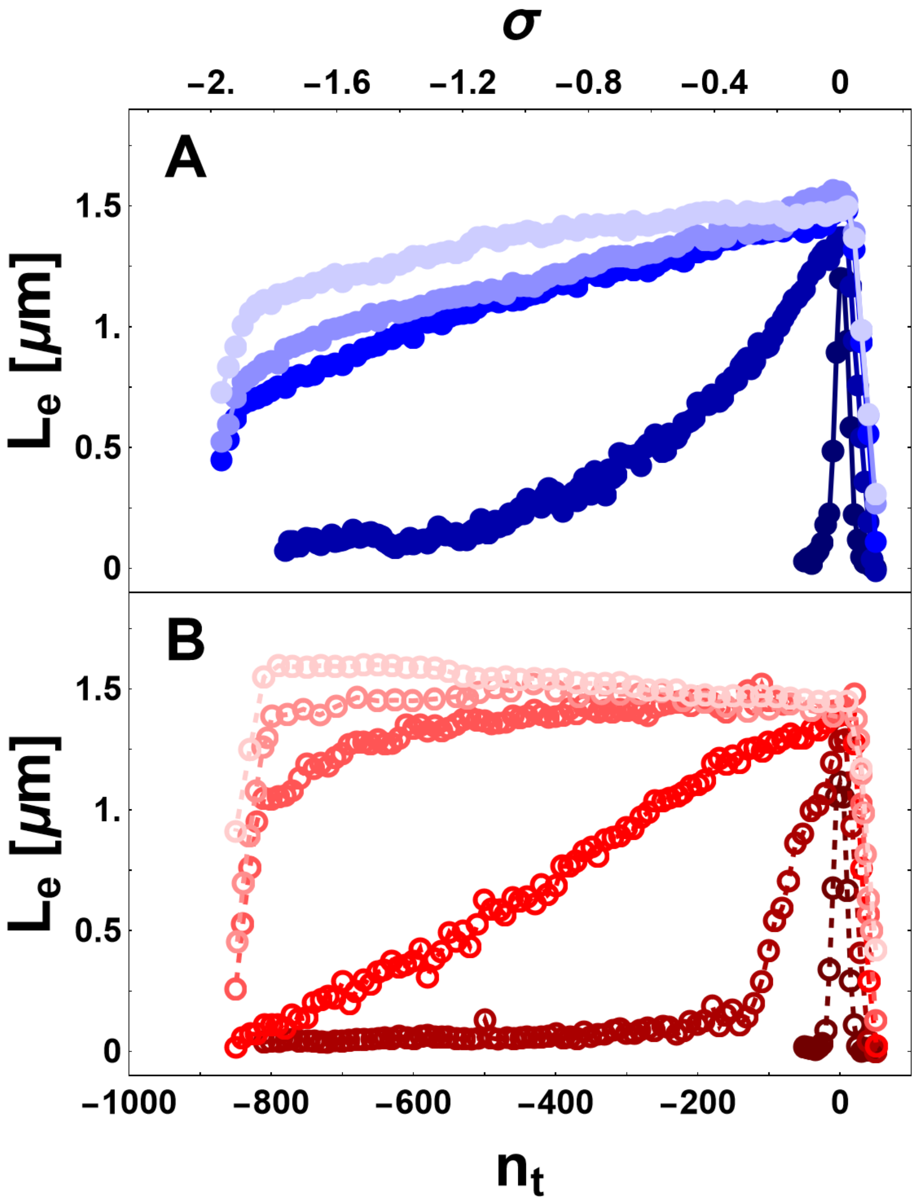

Figure S1. Torsional behavior of WT and DAP DNA. The measured DNA extension, L_e, is plotted as a function of the number of imposed turns, n_t, or equivalently of the supercoiling density, σ, for WT (A, filled circles, at increasing tensions of 0.2, 1.1, 1.7, 2.0, 2.3 pN, from dark to light shades of blue) and DAP DNA (B, open circles, at increasing tensions of 0.2, 0.7, 1.1, 1.7, 2.0, 2.3 pN, from dark to light shades of red).

Mixed Worm Like Chain model

The evaluation of the BL slope is obtained by simply inverting Equations 1 and 2:

$$\frac{L_{ex}}{L_0} = \frac{1}{12}\left((9+4f) + \frac{-9+24f-16f^2}{\left(243-108f+144f^2-64f^3+12\sqrt{3}\sqrt{135-108f+144f^2-64f^3}\right)^{1/3}} - \left(243-108f+144f^2-64f^3+12\sqrt{3}\sqrt{135-108f+144f^2-64f^3}\right)^{1/3} \right)$$

where $f = \frac{FL_{px}}{k_B T}$, $L_{ex} = L_{eB}$ or L_{eL} and $L_{px} = L_{pB}$ or L_{pL}.

Furthermore since in Equation 3 the only n_t dependence is in χ, the value of the BL slope becomes

$$\frac{dL_{eBL}}{dn_t} = \frac{L_{eL} - L_{eB}}{n_{b,max} - n_{t,max}}$$

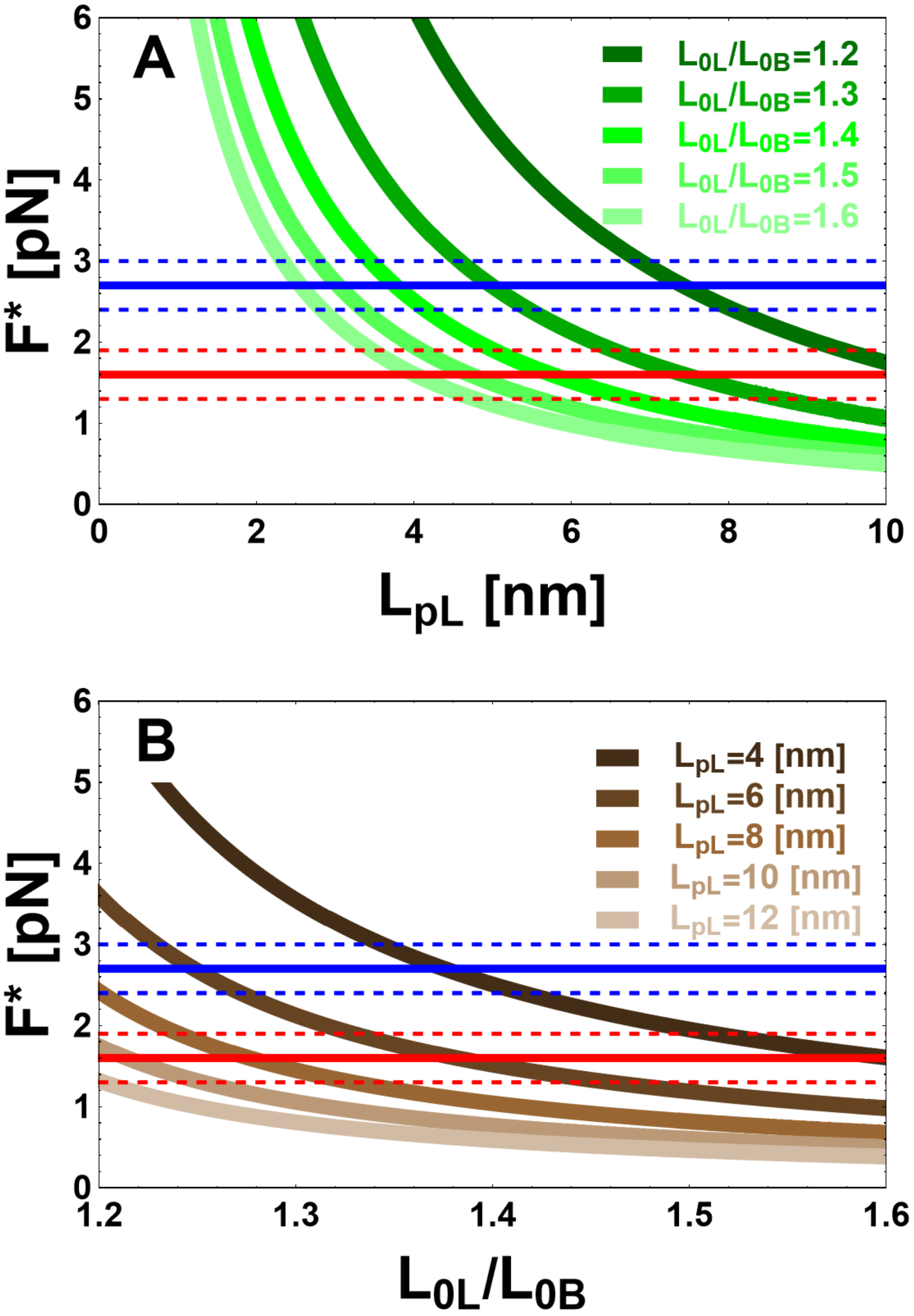

Figure S2. Theoretical values of the inversion force F* calculated as a function of the persistence length L_{pL} of the L phase and ratio L_{0L}/L_{0B} between the DNA L and B extension.

The continuous horizontal lines represent the measured values of the inversion forces F* for WT (blue line) and DAP (red line) DNA. The dashed lines show the uncertainty of the measured forces (F^*_{DAP}=1.6 ± 0.3 pN and F^*_{WT}=2.7 ± 0.3 pN). F* was calculated as a function of L_{pL} for L_{0L}/L_{0B}=1.2, 1.3, 1.4, 1.5, 1.6, (A) or as a function of L_{0L}/L_{0B} with L_{pL}=4, 6, 8, 10, 12 nm (B).

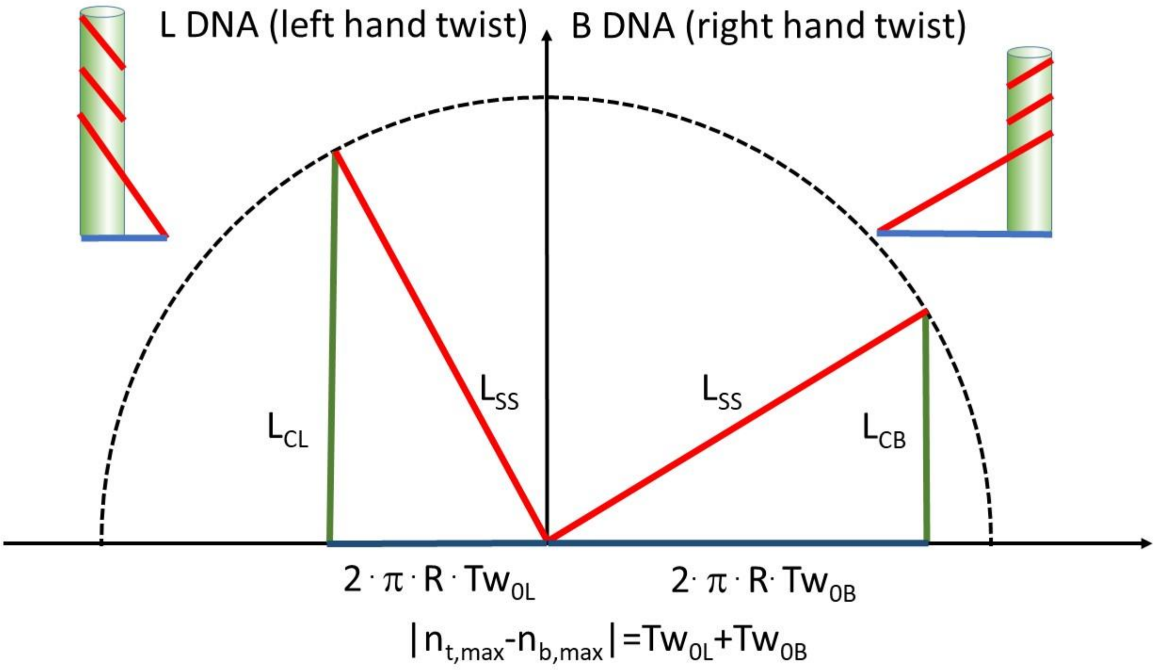

Figure S3. A geometrical model for calculating the contour length increment from B- to L-form DNA. Unwrapping a double-stranded filament, we obtain a triangle (top right for B DNA) and top left for L DNA). The hypotenuse (in red) corresponds to the length of the totally unwrapped filament L=0.6 nm per bp (indicated here as the length of one of the two single strands L_{SS}) which is constant and fixes the locus of the points to which the triangle corner is allowed to belong (dashed circumference). The vertical cathetus (in green) of the triangle corresponds to the contour length of the double stranded DNA, L_{CL} or L_{CB}. The horizontal cathetus (in blue) is determined by the Pythagorean Theorem, and it corresponds to the circumference of the DNA ($2\pi R$, where $R \approx 0.8$ nm is the DNA radius) multiplied the twist of the DNA in the different forms Tw_{0B}=1 turn/10.4 bps and $Tw_{0L} \approx$ 1 turn/14.1 bps. Thus, with constant L, the twist of the DNA form determines its contour length. The relation $|n_{t,max}-n_b|=Tw_{0L}+Tw_{0B}$ imposes the topological constraint on the twist number: the total twist applied to the DNA ($n_{t,max}$) is absorbed as twist by the chain ($n_{b,max}$) which converts the natural twist of the molecule from Tw_{0B} to Tw_{0L}. It follows that since WT and DAP DNA share the same threshold $n_{t,max}$, they also share the same Tw_{0L} and, consequently, the same contour length of the L phase for DAP and WT DNA ($L_{CL,WT}=L_{CL,DAP}$).

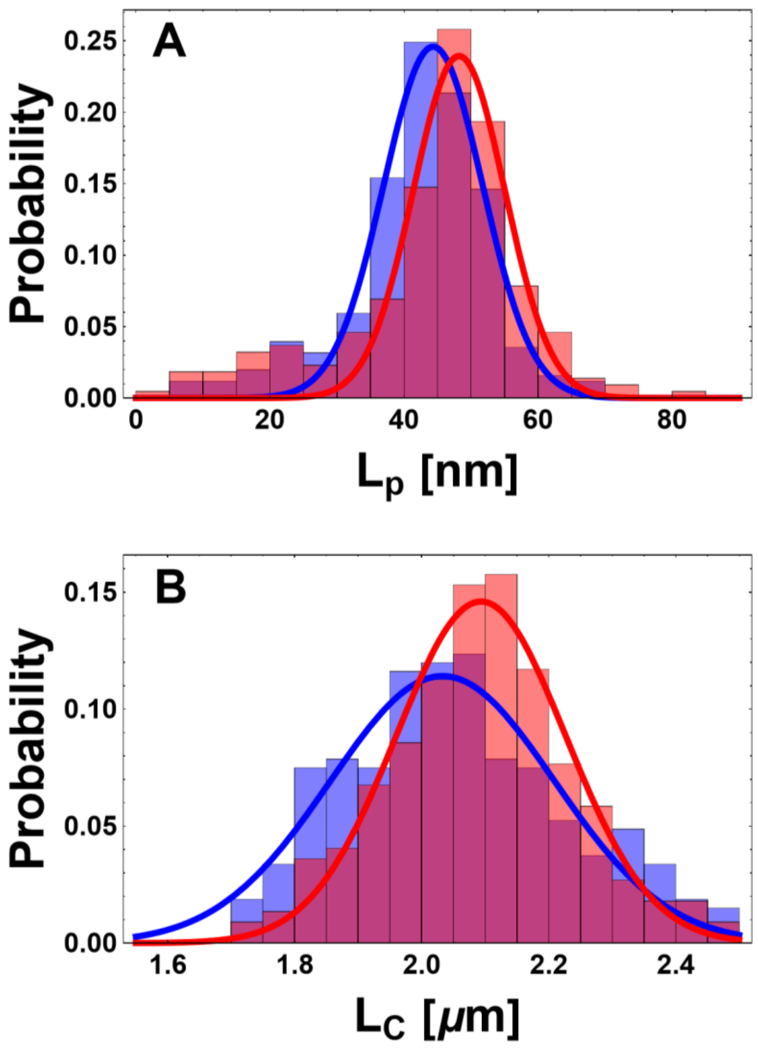

Figure S4: Statistical distribution of the measured DNA persistence length L_p and contour length L_C measured for WT (blue bars) and DAP DNA2 (red bars). Data were obtained by WLC fitting to the L_e vs F data with $L_{CB,WT}$, $L_{pB,WT}$, $L_{CB,DAP}$, and $L_{pB,DAP}$ as free parameters. The continuous lines are gaussian fits for WT data (blue lines) and DAP data (red lines) respectively. The resulting fitting parameters are $L_{CB,WT}$ = 2.0 ± 0.2 μm, $L_{pB,WT}$ = 44.3 ± 7.4 nm, $L_{CB,DAP}$ = 2.1 ± 0.1 μm, $L_{pB,DAP}$ = 48.2 ± 6.8 nm.
